# A systematic review of the scientific evidence of venous supercharging in autologous breast reconstruction with abdominally based flaps

**DOI:** 10.1186/s12957-023-03254-9

**Published:** 2023-12-04

**Authors:** Emma Hansson, Venkat Ramakrishnan, Mary Morgan

**Affiliations:** 1 Department of Plastic Surgery, Institute of Clinical Sciences, The Sahlgrenska Academy, University of Gothenburg, Gröna Stråket 8, 413 45 Gothenburg, Sweden; 2 Department of Plastic Surgery, Region Västra Götaland Sahlgrenska University Hospital, Gröna Stråket 8, SE-413 45, Gothenburg, Sweden; 3 St. Andrew’s Centre for Plastic Surgery and Burns, Broomfield Hospital, Mid Essex Hospital Services NHS Trust, Court Rd, Chelmsford, CM1 7ET Essex UK; 4 St Andrew’s Anglia Ruskin (StAAR) Research Group, Faculty of Health, Education, Medicine & Social Care, Anglia Ruskin University, Chelmsford, UK

**Keywords:** DIEP-flap, TRAM-flap, SIEV, Venous supercharging, Venous augmentation, Plastic surgery procedures, Breast reconstruction, Intraoperative complications

## Abstract

**Background:**

Abdominally based free flaps are commonly used in breast reconstruction*.* A frequent complication is venous congestion, which might contribute to around 40% of flap failures. One way to deal with it is venous supercharging. The primary aim of this study was to investigate the scientific evidence for the effects of venous supercharging.

**Methods:**

A systematic literature search was conducted in PubMed, CINAHL, Embase, and Cochrane library. The included articles were critically appraised, and certainty of evidence was assessed using the Grading of Recommendations, Assessment, Development and Evaluations (GRADE) approach.

**Results:**

Thirty-six studies were included. Most studies had serious study limitations and problems with directness. Three studies report ‘routine’ use of venous supercharging and performed it prophylactically in patients who did not have clinical signs of venous congestion. Seventeen studies report on flap complications, of which one is a randomised controlled trial demonstrating statistically significant lower complication rates in the intervention group. The overall certainty of evidence for the effect of a venous supercharging on flap complications, length of hospital stay and operative time, in patients without clinical signs of venous congestion, is very low (GRADE ⊕ ⊕ ⊝ ⊝), and low on and surgical takebacks (GRADE ⊕ ⊕ ⊝ ⊝). Twenty-one studies presented data on strategies and overall certainty of evidence for using radiological findings, preoperative measurements, and clinical risk factors to make decisions on venous supercharging is very low (GRADE ⊕ ⊝ ⊝ ⊝).

**Conclusion:**

There is little scientific evidence for how to predict in which cases, without clinical signs of venous congestion, venous supercharging should be performed. The complication rate might be lower in patients in which a prophylactic venous anastomosis has been performed.

**Trial registration:**

PROSPERO (CRD42022353591).

**Supplementary Information:**

The online version contains supplementary material available at 10.1186/s12957-023-03254-9.

## Introduction

Nowadays, the usage of abdominally based free flaps is considered one of the standard techniques to reconstruct breasts [1]. It was first described in 1979 and popularised as the transverse rectus abdominus muscle (TRAM)-flap, including the muscle, and later developed to a muscle sparing technique (ms-TRAM) and to a technique not sacrificing any muscle at all, deep inferior epigastric perforator (DIEP)-flap [2–7]. One of the pitfalls of abdominally based free flaps is venous congestion, which occurs in 2 to 15% of flaps and it has been estimated that venous congestion contributes to around 40% of flap failures [8–11]. Venous congestion can be caused by both surgical factors, such as anastomotic or vessel failure, and poor perforator selection, as well as anatomical factors, principally superficially dominant flap drainage which makes the deep inferior epigastric vein insufficient to drain the flap [12].

The abdominal free flaps used for breast reconstruction are most often based on the deep inferior epigastric artery, arising from the external iliac artery, which is the most significant supplier of the skin and subcutaneous tissue of the abdominal wall, the *source artery* to the *angiosome* [13, 14]. The abdominal cutaneous territory of the superficial epigastric artery is smaller [13]. Contrary to this, the venous drainage of the skin and subcutaneous tissue of the lower abdomen is primarily via the superficial venous system and secondarily via the deep venous system. As the main pedicle of the abdominally based flaps is part of the deep system, a working flap drainage is dependent on veins interconnecting the deep and superficial venous system and can explain some of the venous congestion sometimes seen in flaps with a patent deep epigastric vein anastomosis [15–18]. It was recognised already in the early publications; for example, by Hartrampf et al., who described that he tried to improve venous drainage by ligating the ipsilateral deep inferior epigastric vessels in 3 of 8 cases to achieve an opening of connections between the deep and superficial venous system before the flap was raised [3].

Anastomosis of the superficial inferior epigastric vein (SIEV) to a recipient vessel, and thereby connecting the superficial venous system, is a well-described technique to increase the amount of tissue that can be transferred, to salvage postoperatively congested DIEP flaps, and to resolve intraoperative venous congestion, when a single-vein anastomosis of the deep inferior epigastric system is not sufficient [19–24]. The technique is called *venous supercharging*, *augmentation,* or *super-drainage.* Nonetheless, there is no clear consensus regarding how the SIEV should be anastomosed, if it should be performed prophylactically, and if it can be predicted in which cases the superficial venous system must be anastomosed to achieve a working flap drainage.

The primary aim of this study was to investigate the scientific evidence for how and when venous supercharging should be performed in autologous breast reconstruction with an abdominally based free flap.

## Methods

### Protocol

This is a systematic review of the evidence for the usage of SIEV for venous supercharging in autologous breast reconstruction with a DIEP-flap. The protocol was pre-registered in PROSPERO (CRD42022353591) (https://www.crd.york.ac.uk/prospero/display_record.php?RecordID=353591) and reported according to the PRISMA guidelines [25]. The PRISMA checklist is included in the Additional files.

### Eligibility criteria

**Fig. 1:**
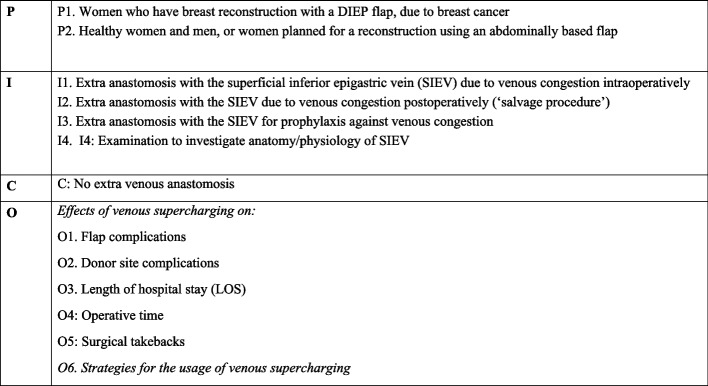
*PICO* patients interventions comparison outcomes

Inclusion criteria were studies examining the effects of the usage of SIEV and strategies for when it should be used. Narrative and systematic review, textbooks, comments, and case reports describing aspects that have been included in other studies were excluded. Included article had to meet criteria defined in a PICO (Population, Intervention, Comparison, and Outcome) [26], which is described in Fig. 1. The authors independently assessed if the articles met the inclusion criteria and disagreements were resolved by discussion.

### Information sources, search strategy and selection process

PubMed, CINAHL (EBSCO), Embase, and Cochrane library databases were searched for articles and abstracts published before 18.09.2023, the date when the search was conducted. The search string was (((DIEP) OR (deep inferior epigastric perforator)) OR (breast reconstruction)) AND (((superficial inferior epigastric) OR (SIEV)) OR (superficial venous)) in PubMed, CINAHL and Cochrane library and (‘diep’ OR ‘deep inferior epigastric perforator flap’/exp OR ‘deep inferior epigastric perforator flap’) AND ‘superficial inferior epigastric’ in Embase. Moreover, all bibliographies of included studies were manually checked. The search was limited to studies published in English, French, German, Italian, Swedish, Danish, and Norwegian. When eligibility for inclusion could not be assessed with the information in the abstract, the entire article was read and assessed.

### Data collection process and data items

The authors collected data from the articles independently and collected in a Microsoft Excel (Microsoft Corporation, Redmond, Washington, USA). Information collected included first author, year of publication, study country, study design, study objective, number of included participants and controls, interventions, definition of venous congestion, and on the outcomes defined in the PICO (Fig. 1). In the table of included studies (Table 1), the study design of the study is given. In the outcome tables, the study design used to study that particular outcome is stated. During the collection processes, it became clear that some outcomes had to be subdivided into different themes to allow for interpretation and synthesis of the information. Strategies were subdivided into pre-operative radiological findings, intraoperative measurements and clinical signs.

**Table 1:** Included studies

**Author** **Year** **Country**	**Scope of study**	**Study design**	**Population (n)**	**Study groups; Intervention and control (*n* = no. of DIEPs/TRAMs)**	**Definition of venous congestion**	**Recipient vessel**	**Outcomes**	**Directness**^a^	**Study limitations**^a^	**Precision**^a^
Al-Dhamin, Canda 2014 [27]	Evaluation of the retrograde limb of the internal mammary vein as a second recipient vein	Non-randomised study (retrospective) with controls	P1: 48 DIEPs (38 pats)	I1: 17 C: 31	NR	The DIEV was anastomosed to the proximal IMV and the SIEV was anastomosed to the distal IMV	O1. Flap complications	−	+ ?	−
Al Hindi, 2019, France [28]	Analyse the centre’s use of SIEVs	Non-randomised study (retrospective) with controls	P1: 198 DIEPs (183 pats)	I1: 15 I2: 2 C: 181	Blueish discoloration and increased capillary refill time (> 3 s)	SIEV 11/17 Second DIEV 6/17 Recipient vessels: CV 8 IMV 4 LMV 2 CSV 2 TDV 1	O1. Flap complications O3. LOS O4. Operative time	-	-	-
Ali, 2010, Taiwan [29]	Investigation of compilations in intraoperatively congested DIEPs	Non-randomised study (retrospective) with controls	P1: 151 DIEPs	I1: 14 I2: 7 C: 130	‘A purplish or plethoric coolness of the flap associated with brisk capillary refill (< 1 s) and rapid and dark venous bleeding on puncture, despite a patent venous anastomosis’	SIEV Second DIEV Recipient vessels: IMV EJV TDV ± VG	O1. Flap complications O3. LOS	−	−	?
Akita, 2018, Japan [30]	Pilot-testing of a method to detect venous congestion intraoperatively	Non-randomised study (prospective) with controls	P1: 70 DIEPs (67 pats)	I1: 8 C: 62	NA	Ipsilateral SIEV to LTV (*n* = 3) DIEV (*n* = 2)	O6. Strategies	−	+ ?	?
Ayestaray, 2016, France [31]	Investigation of the effect of venous supercharging on complication	RCT	P1: 52 DIEPs	I3: 29 C: 23 *Patients randomised to supercharging or not supercharging*	NR	Ipsilateral SIEV to TAV	O1. Flap complications O4. Operative time O5. Operative takebacks	?	+	−
Bartlett, 2018, USA [32]	Description of centre’s algorithm for intraoperative salvage of congested flaps	Case series (retrospective)	P1: 67 DIEPs (38 pats) C: 172 DIEPs (100 pats)	I1: 40	• Brisk capillary refill (< 1.5 s) • Red/purple hue • Dilated/ tense SIEV • Brisk bleedning during flap trimming or de-epithelialisation	32 SIEV to DIEV 5 SIEV to IMV	O1. Flap complications O4. Operative time	−	−	−
Bast, 2016 [33]	Investigation of the ratio of the sub- and supra-scarpal fat layers, the number of deep system perforators, and of the SIEV diameter and if indications for SIEV dissection can be predicted by these factors	Case series (retrospective)	P2: 50 women (100 hemiabdomens)	I4: 50 CTA	NA	NA	O6. Strategies	−	+ ?	?
Beier, 2013, German [34]	Investigation of assess the potential role for intra-operative CLDS for intra-operative decision-making	Case series (prospective)	P1: 25 DIEPs or muscle sparing TRAMs	I4: CTA (preop), Combined lased Doppler spectrophotometry (CLDS) (intraop)	NA	NA	O6. Strategies	−	+ ?	?
Blondeel, 2000, Belgium [9]	Comparison of venous congestion in DIEPs vs. TRAMs, suggestion of strategy for dealing with venous congestion and investigation of why venous congestion occur more frequently in zone IV of a DIEP flap	Non-randomised study (retrospective) with controls and case series (prospective) of cadavaers	P1: 249 DIEPs (214 pats)	I1: 5 C: 245	‘Severe diffuse venous congestion that involved the whole flap. Particularly large SIEV (> 1.5 mm)’	NR	O6. Strategies	−	+ ?	?
Boutros, 2013, USA [35]	Investigate the outcome of the centre’s routine use of venous augmentation	Non-randomised study (retrospective) with controls	P1: 352 DIEPs (192 pats)	I3 (?): 311 C: 42 *NR how the patients were allocated to the two groups. Authors describe their use of venous supercharging was routinely used*	Ultrasound diagnosis	The largest superficial vein to the more medial IMV or to the perforator of IMV	O1. Flap complications O5. Surgical takebacks	−		−
Davis, 2018, UK [18]	Correlation of preoperative CTA findings with postoperative venous congestion to predict patients at risk of congestion	Non-randomised study (prospective) with controls	P1: 240 DIEPs	I1 + 2: 13 C: 227 I4: CTA	‘Sufficient clinical evidence necessitating a salvage procedure’	SIEV to DIEV (SOS-technique) Retrograde IMV Second *v. comitantis* of IMV CV TV	O6. Strategies	−	+ ?	?
Dortch, 2018, USA [36]	Investigate venous characteristics associated with SIEV augmentation	Non-randomised study (retrospective) with controls	P1. 99 DIEPs or muscle sparing TRAMs	I1: 29 I4: Ferumoxytol-enhanced magnetic resonance angiography C: 73	NR	NR	O6. Strategies	−	+ ?	?
Enajat, 2010, Australia [37]	Comparison of one and two veins for drainage	Non-randomised study (retrospective) with controls	564 DIEPs (501 pats)	I1: 291 C: 273	‘Brisk capillary refill or bleeding or deep blue colour of the flap or draining blood’	SIEV (92%) or second DIEV (8%) to CV 83% IMV 10% CSV 4% TDV 4%	O1. Flap complications O4. Operative time			−
Eom, 2011, South Korea [38]	Review of centre’s experiences and comparison of different recipient veins	Non-randomised study (retrospective) with controls	P1: 153 DIEPs and TRAMs	I2: 45 C: 108	‘Signs of congestion’	SIEV to 31 LTVs 10 branches of TAV 4 perforator of IMV The contralateral SIEV was used as VG in 4 cases	O1. Flap complications O5. Take backs	−	−	−
Galanis, 2014, USA [39]	Description of centre’s strategies to handle intraoperative venous congestion	Description of approach	NA	NA	NA	NA	O6. Strategies	−	−	−
Huang, 2022, USA [40]	Investigation of risk factors for venous congestion	Non-randomised study (retrospective) with controls	455 DIEPs (258 pats)	I1: 5 I2: 3 I4: CTA C: 447	‘Flap engorgement and colour change’	NR	O6. Strategies	?	+ ?	?
Katz, 2010, USA [41]	Classification of clinically relevant CTA scenarios	Case series (prospective)	P2: 172 hemiabdomens (86 pats)	P4: CTA	NA	NA	O6. Strategies	−	+ ?	?
La Padula, 2016, France [42]	Assesment of the retrograde IMV as a recipient vessel	Non-randomised study (retrospective) with controls	P1: 74 DIEPs	I2: 36 C: 38	Clinical signs of venous congestion	SIEV/second DIEV to retrograde limb of IMV	O1. Flap complications O3. LOS O5. Operative takebacks	−	+ ?	?
Lee, 2013, South Korea [43]	Evaluation of the association between ischaemic time and fat necrosis in DIEP	Non-randomised study (retrospective) with controls	P1: 86 DIEPs	P1: 18 C: 68	NR		O1. Flap complications	−	?	?
Lundberg, 2006, Sweden [44]	Evaluation of 50 consecutive DIEPs to develop a strategy to avoid complications	Non-randomised study (retrospective) with controls	P1: 50 DIEPs	P1:3 P2: 3 C:44	Capillary refill < 2 s	SIEV to CV	O6. Strategies	−	−	−
Nedomansky, 2018, Austria [45]	Evaluation whether SIEV dissection increases the risk for abdominal seroma	Non -randomised study (retrospective) with controls	P1: 100 DIEPs	39 pat with SIEV dissection (29 unilateral and 10 bilateral) C: 61	‘Livid discoloration of the flap in combination with an erected SIEV stump’	NR	O2. Donor site complications O3. LOS	−	? +	?
Ochoa, 2013, USA [46]	Determination of the incidence of flap morbidity following venous augmentation among intraoperatively congested DIEP flaps	Non-randomised study (retrospective) with controls Narrative review	P1: 87 DIEPs (81 patients)	I1: 87 DIEPs (81 pats) C: 629 DIEPs (418 pats)	‘Brisk capillary refill. Cutaneous discoloration that improves promptly when release of venous blood through the SIEV’	59 SIEV 28 DIEV To 50 IMV 24 IMV perforators 6 DIEV 5 LTV 2 TDV 15 VG	O1. Flap complications O3. LOS O4. Operative time O6. Strategies	−	−	−
Rothenberger, 2013, Germany [47]	Investigate the venous drainage of the DIEP flap	Case series (prospective)	P1: 19 DIEPs	I4: Clamping of ipsilateral and contralateral and both SIEVs intraoperatively to determine the efficiency of venous outflow	NA	NA	O6. Strategies	−	+ ?	?
Rubino, 2009, Italy [48]	‘Investigation of the correlation between flow rate and size in perforator flaps and, estimation of the minimum diameter of the perforating vein needed to drain flaps of given weights.’	Case series (prospective)	P1: 19 DIEPs	I4: Echo-colour-Doppler	NA	NA	O6. Strategies	−	+ ?	?
Xin, 2012, China [49]	Investigation of the efficacy of venous augmentation Presentation of a new technique	Non-randomised study (retrospective) with controls	P1: 79 DIEPs	I1: 32 C: 47 I4: Blood pressure measurements in anastomoses	Large flaps and/or clinical signs	SIEV to TDV, LTV, IMV, or DIEV	O1. Flap complications O4. Operative time	−	−	−
Sadik, 2013, USA [50]	Definition of predictors for a superficially dominant venous system	Non-randomised study (prospective) with controls	P1: 39 DIEPs	I1: 6 C: 33 I4: CTA—measurement of the SIEV diameter preoperatively. The diameter was also measured intraoperatively	NR	NR	O6. Strategies		+ ?	?
Santanelli, 2015, Italy [51]	Investigation of predictive and protective factors for perfusion related complication	Non-randomised study (retrospective) with controls	P1: 287 DIEPs	I1: 173 C:74	NR	NR	O1. Flap complications	−	+ ?	?
Schaverien, 2010, UK [52]	Investigation of the relationship between presurgical venous pattern analysed with MRA and venous compromises	Case series (retrospective)	P1: 54 DIEPs	I4: Preoperative contrast-enhanced magnetic resonance angiography	NR	NA	O6. Strategies	−	+ ?	?
Smit, 2010, Sweden (multicentre) [53]	Investigation of pressure in the SIEV before and after flap dissection and correlation to venous congestion	Case series (prospective)	P1: 26 DIEPs	I4: Measurement of venous pressure	NR	NR	O6. Strategies	−	+ ?	?
Svee, 2023, Sweden [54]	Investigation of arm lymphoedema after the usage of CV	Case series (retrospective)	P1: 54 DIEPs	I1: 27 C: 27	NR	SIEV to CV	O2. Donor site morbidity	−	+ ?	+ ?
Tokumoto, 2019, Japan [55]	Investigation of whether CV is equivalent to SA and LTV for superdrainage	Non-randomised study (retrospective) with controls	P1: 88 DIEPs (88 pats)	I1: 45 C: 43	Clinical signs of venous congestion and/or bleeding from the SIEV after anastomosis	SIEV to 22 SA 16 LT 7 CV The extra anastomosis was performed in patients where the IMV diameter was smaller than that of the DIEV	O1. Flap complications O2. Donor site complications O4. Operative time O5. Operative take backs O6. Strategies	−	−	?
Unukovych, 2016, Sweden [56]	Evaluate perioperative predictors of complications and reoperations in DIEP	Case series (retrospective)	P1: 503 DIEPs (433 pats)	I1: 211 C: 292	‘Discretion of surgeon’		O1. Complications	−	?	?
Varnava, 2023, Germany [57]	Review of centres use of SIEV	Case series (retrospective)	P1: 150 DIEPs (107 pats)	I1: 4 C: 146	NR	NR	O1. Complications O5. Operative take backs	−	−	−
Vijayasekaran, 2017, USA [58]	Analyse of the centres use of SIEV and retrograde IMV Description of algorithm for routine use of SIEV	Non-randomised study (retrospective) with controls *Routine venous augmentation -two consecutive cohorts*	P1: 60 DIEPs	I3: 30 C: 30 *Two consecutive series*	NA	SIEV to the retrograde IMV	O1. Flap complications O5. Operative take backs O6. Strategies	?	+	?
Wagels, 2015, Australia [59]	Investigation of signs on CTA that can predict venous congestion	Non- randomised study (retrospective) with controls	P1: 124 DIEPs/TRAMs (96 pats)	I4: CTA	NA	NR	O6. Strategies	−	+ ?	?
Zhu, 2023, Republic of Korea [60]	Investigation of the clinical efficacy of the use of SIEV using indocyanine green intraoperatively	Non- randomised study (retrospective)	P1: 62 ms-TRAMS, 6 DIEPs	I4: CTA and indocyanine green	NA	NR	O6. Strategies	−	+ ?	?

^a^ + No or minor problems, ? some problems, − major problems

### Risk of bias in individual studies and across studies

The included studies were critically appraised using checklists modified from the Swedish Agency for Health Technology Assessment and Assessment of Social Services (*SBU*) [61]*.*

The overall certainty of evidence was classified as very low (GRADE ⊕ ⊝ ⊝ ⊝), low (GRADE ⊕ ⊕ ⊝ ⊝), moderate (GRADE ⊕ ⊕ ⊕ ⊝), or high (GRADE ⊕ ⊕ ⊕ ⊕) according to the GRADE system (Grading of Recommendations, Assessment, Development and Evaluations) [62]. Factors determining the quality of evidence are described under the headline ‘risk of bias within studies and across studies’ in the results section.

## Results

### Study selection

**Fig. 2:**
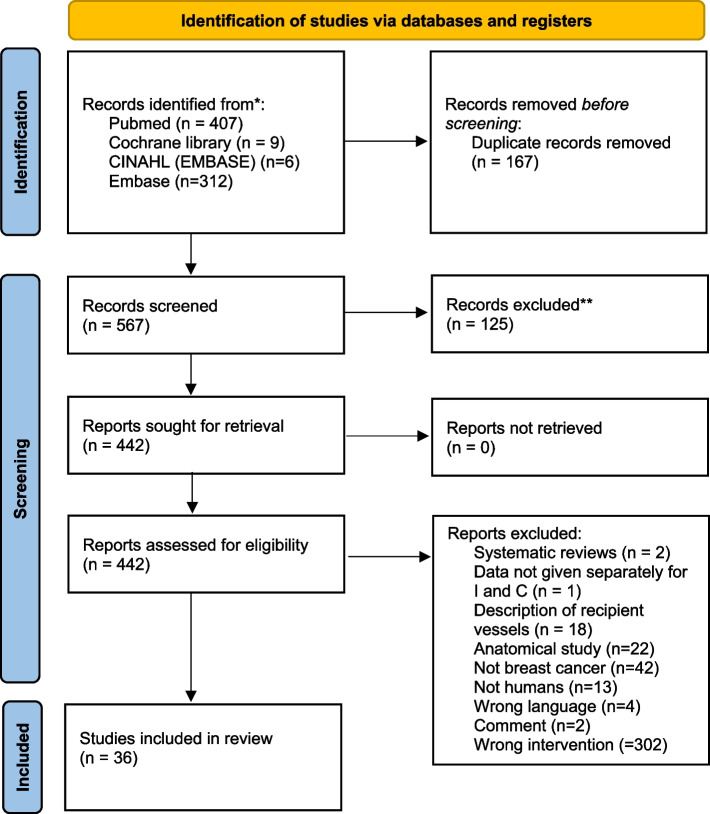
PRISMA diagramme

A total of 567 abstracts were retrieved following the searches, when duplicates had been removed (Fig. 2). Of these, 125 did not meet the inclusion criteria and were excluded, leaving 442 articles that were read in full text. After a more detailed scrutiny, a further 406 articles were excluded, resulting in 36 studies to be included in the review (Table 1).

### Study characteristics

Effects of venous supercharging were studied mainly in non-randomised cohorts with controls, where venous supercharging was performed in case of signs of venous congestion intraoperatively or postoperatively. Three studies report ‘routine’ use of venous supercharging and performed it prophylactically in patients who did not have clinical signs of venous congestion. One of the studies randomised patients to venous supercharging or not supercharging (29 vs. 23 pats) [31], one reported two consecutive series comprising 30 pats in each group [58] and one did not state how the patients were allocated to the two groups [35]. For the randomised controlled trial (RCT), a sample size calculation was not performed and primary and secondary end points as well as outcome measures were not defined.

### Flap complications

Seventeen studies report on flap complications [27–29, 31, 32, 35, 37, 38, 42, 43, 46, 49, 51, 55–58], of which one is an RCT [31] and two are controlled cohort studies [35, 58] including prophylactic venous supercharging in patients without clinical signs of venous congestion (Additional file 1). The groups of the RCT [31] and the study comparing two consecutive cohorts [58] have groups that are comparable with regards to age, body mass index (BMI) and comorbidities (Additional file 1). The three studies share the methodological weakness that they have not defined complications and how, when and by whom they were diagnosed. The RCT [31] demonstrated statistically significant lower complication rates in the intervention group. The findings were similar in the two cohort studies [35, 58]. Nonetheless, the magnitude of the effect on complications of a SIEV must be interpreted with caution as the samples are small and there is a clear heterogenicity in the frequency of complications. Venous congestion was 13% in the intervention group and 55% in the control group and partial flap loss 9% in the intervention group and 45% in the control group in the RCT [31]. Total flap loss was 0 in all three intervention groups and ranged from to 0% [35, 58] to 17% [31] in the control groups. The numbers of the studies comparing two incomparable groups, patients with and without clinical signs of venous congestion, are given in Additional file 1.

The overall certainty of evidence for the effect of a SIEV anastomosis on flap complications, in patients without clinical signs of venous congestion, is low (GRADE ⊕ ⊕ ⊝ ⊝). The evidence was downgraded three levels due to a high risk of bias, imprecision and inconsistency in the magnitude of effect and upgraded one level as magnitude of the effects of venous supercharging on flap complications seems to be large and is consistent across studies.

### Donor site complications

Three retrospective non-randomised studies with controls reported donor site complications (Additional file 2) [45, 54, 63]. One of the studies [55] reported that there were zero donor site complications, whereas the other [45] stated that the rate of abdominal seromas requiring drainage was higher in the group where the SIEV had been harvested, especially if it had been harvested bilaterally. However, the study did not control for other factors that might increase the rate of seromas. The third study reported that the usage of the cephalic vein as a recipient vessel does not seem to increase the risk for arm lymphoedema [54].

The overall certainty of evidence for the occurrence of donor site complications after SIEV harvesting is very low (GRADE ⊕ ⊝ ⊝ ⊝). The evidence was downgraded three levels due to a very high risk of bias, indirectness, and imprecision.

### Length of hospital stay

Five retrospective non-randomised controlled cohort studies reported LOS [28, 29, 42, 45, 46]. Most of the studies stated a longer LOS in the venous supercharging group than among the controls (Additional file 3). Nonetheless, the studies did not have comparable groups and did not control for confounders that might affect LOS, such as both patient related and surgical factors as well local tradition.

The overall certainty of evidence for the effect of venous supercharging on LOS is very low (GRADE ⊕ ⊝ ⊝ ⊝). The evidence was downgraded three levels due to a high risk of bias, indirectness, and imprecision.

### Operative time

Seven studies reported operative time, of which one was an RCT and six were retrospective non-randomised controlled cohort studies [28, 30–32, 37, 46, 49]. Most of the studies stated a longer operative time in the venous supercharging group than among the controls (Additional file 4). The RCT demonstrated that the mean time increased from 405 to 510 min [31]. However, all the included studies had methodological flaws such as lack of information on learning curves and techniques used and in case of the retrospective studies, different populations among the interventions and the controls and no adjustment for confounders that might affect operative time. As only patients with clinical signs of congestion were included in the venous supercharging group, it is likely that aspects, other than the SIEV dissection and anastomosis itself, also affected operative time. Moreover, none of the studies took possible savings in re-operation times into considerations.

The overall certainty of evidence for the effect of venous supercharging on the operative time is very low (GRADE ⊕ ⊝ ⊝ ⊝). The evidence was downgraded three levels due to a very high risk of bias, indirectness, and imprecision.

### Surgical takebacks

Seven studies reported surgical take backs of which one was an RCT [31] and six retrospective non-randomised controlled cohort studies (Additional file 5) [31, 35, 38, 42, 55, 57, 58]. In the RCT [31] and the study with two consecutive series [58], there were considerable differences in takebacks between the groups, 13% vs. 55% and 1% vs. 10%, respectively. The other studies also consistently showed that the takeback rates were lower in the intervention groups. However, the magnitude of the decrease of takebacks is unclear as intervention and control groups were completely different in some studies which could result in an underestimation of takebacks. In brief, the magnitude of the effect of venous augmentation on surgical takebacks is unclear due to uncompilable groups in many studies and small samples in the more high-quality studies.

The overall certainty of evidence for the effect of venous supercharging on surgical takebacks is low (GRADE ⊕ ⊕ ⊝ ⊝). The evidence was downgraded three levels due to a high risk of bias, imprecision and indirectness and upgraded one level as magnitude of the effects of venous supercharging on surgical takebacks seems to be large and is consistent across studies.

### Strategies for the usage of venous supercharging

Twenty-one studies presented data on strategies for when venous supercharging should be performed (Additional file 6) [9, 18, 30, 33, 34, 36, 39–41, 44, 46–48, 50, 52, 53, 55, 58–60]. Nine [18, 33, 36, 40, 41, 50, 52, 59, 60] of them present radiological signs that could predict venous congestion, four intra-operative measurements [30, 34, 47, 53] and six [9, 39, 46, 48, 50, 55] clinical signs that could predict the need for venous supercharging. Two studies [44, 58] merely give recommendations based on their clinical experience. The studies investigation predictors generally compare patients who have had clinical venous congestion with those that have not and can thereby be classified as non-randomised observational studies with controls.

Regarding radiological findings predictive of venous supercharging, a few studies have investigated the role of the SIEV diameter. One study concluded that a big SIEV diameter or deep inferior epigastric vein (DIEV) diameter and a high SIEV/DIEV diameter ratio (no cut off values are given) [36] and another that SIEV size > DIEV size at origin (5.2 vs 3.5 mm, *p* = 0.007) [59] were predictive of the need for supercharging, whereas two other found that the diameter seems to be negatively correlated to the need for venous supercharging [40] and that the was no correlation between SIEV diameter and the need for supercharging [50]. Four studies investigated connection between the deep and superficial system on computed tomography (CT) and concluded that venous supercharging is needed when there are no direct [52, 59] or atypical connections (in terms of caliber, tortuosity or superficial path) [18] between the superficial and the deep system radiologically or signs of a superficially dominant system [41] or an axial non-arborising superficial system [59]. The thickness of the suprascarpal fat pad on CT has given rise to contradictory results as one study [33] found that a suprascarpal fat pad thickness of > 23 mm and another [40] that a suprascarpal fat pad thickness of < 18 mm is predictive of the need for venous supercharging. In brief, branching patterns have consistently shown to be predictive of the need for venous supercharging, whereas there have not been any consistent findings for SIEV size and suprascarpal fat pat thickness.

A potential intraoperative measurement that could be used include the ratio of blood glucose content in the flap to systemic blood glucose, where a low index seems to be predictive of venous congestion [30]. Similarly, the relative haemoglobin concentration in the flap also seems predictive of the need for supercharging [47], as well as a pressure increase in the SIEV [53]. One centre has used a combined laser Doppler spectrophotometry system, which seemed to be helpful in some cases that were not clearly congested clinically [34]. The four studies on intraoperative measurements that could predict the need for venous supercharging have all studied different methods in small samples and must be considered pilot studies.

Suggested clinical risk factors for the need for venous augmentation are a SIEV diameter of > 1.5 mm [9], a bigger flap [48], previous abdominal surgery (odds ratio (OR): 0.8 (0.66–0.99), *p* = 0.03) [46] and a high BMI (OR: 10.4 (0.99–1.10), *p* = 0.14) [46]. The latter has been contradicted by a study showing no correlation between the need for venous augmentation and BMI or the BMI:SIEV size ratio [50]. In brief, there are no risk factors, that have been scientifically validated clinical and adjusted for confounders, that can used to predict the need for venous supercharging.

The overall certainty of evidence for using radiological findings, preoperative measurements and clinical risk factors to decide whether to use venous supercharging is very low (GRADE ⊕ ⊝ ⊝ ⊝). The evidence was downgraded three levels due to a very high risk of bias, indirectness, and imprecision.

### Risk of bias within studies and across studies

A summary of the evaluation of individual studies is given in Table 1. The studies had serious study limitations (*risk of bias*) affecting the quality of evidence. Regarding study design, only one RCT [31], providing high-quality evidence, could be included in the review, whereas the others were observational, providing low quality evidence. Among the other two studies that investigated ‘routine’ use of venous supercharging at least one [58] of the two studies with two consecutive series can be regarded as ‘quasi’ or ‘pseudo’ randomised as it allocated patients according to when the patient was operated. In the other studies, the patients were allocated to intervention or control based on subjective clinical signs of venous congestion and at the ‘discretion of the surgeon’ and therefore it can be presumed that the exposed and the unexposed patients were selected from different populations, one with a higher risk of complications due to venous congestion than the other. This makes the groups incomparable.

None of the surgeons who performed the operations were, for natural reasons, blinded to the allocation of patients. None of the studies state if the outcome assessors of complications were blinded. It is less important for outcomes such as total flap failure but might influence less defined surgical outcomes such as fat necrosis. All the studies have an incomplete account of the outcome events, that is definition of complications and how and when they were diagnosed, surveyed and registered, which makes the outcome measures unvalidated and probably varying both within and across studies. Moreover, there are several other aspects than venous supercharging that might affect the outcomes, both patient-related and related to the operation, and most of the studies had not controlled for these and therefore there are a lot of potential confounding factors.

For several outcomes, there was a clear *inconsistency*, an unexplained heterogeneity of results, for example in the magnitude of treatment effect of venous supercharging. This is probably explained by different baseline risks (different patients among the exposed and the non-exposed) and different definitions of the outcomes and different follow-up times. The small samples could also have contributed.

Looking at sources of *indirectness*, we can conclude that there is a limitation of the applicability of the results as the populations in the different studies probably vary considerably. Moreover, the delivery of interventions, when venous supercharging was performed, by whom and in which patients probably also vary considerably across studies. None of the studies have defined primary and secondary outcomes and none specified which outcomes are the most important to the patients. However, most of the studies use outcomes that we can assume are important for the patients, such as flap loss and length of hospital stay, and few use surrogate outcomes. Moreover, most of the studies make direct comparisons between venous supercharging and not venous supercharging, albeit it in non-comparable groups.

All of the studies suffer from *imprecision* as small samples were included. The studies with a somewhat lower risk for bias included between 20 and 30 patients in the different groups [31, 58], which has to be considered few. In many of the studies there were few cases of clinical venous congestion and thereby of intervention. Moreover, there were few serious adverse advents, such as total flap loss, in the studies, which also makes the results less reliable.

## Discussion

The usage of venous supercharging in abdominally based autologous breast reconstruction is a matter of continuous clinical discussion. This systematic review has identified studies on venous supercharging on different aspects. The scientific quality of the existing studies is weak and further high-quality studies are necessary to evaluate the effects of routine use of it.

The potential benefit of routine use of venous supercharging could be a lower rate of venous congestion requiring surgical intervention and of consequent complications [20, 21]. Venous supercharging could potentially increase the safety of abdominally based free flaps as venous congestion is a significant cause of total flap failures [11]. It could be particularly useful for example in frail patients [64]. Some authors have concluded that the only disadvantage of routinely performed venous supercharging would be ‘an increase of operative time of 20 min and the cost of an extra coupler [COUPLER^©^ device (Synovis Micro Companies Alliance, Inc. Birmingham, AL, USA)]’ [58]. Nonetheless, this review demonstrates that there are other potential drawbacks, such as potential donor site complications where the SIEV has been dissected as well as where the recipient vessel has been dissected, that should be considered [45].

It seems straightforward that abdominally based flaps that are superficially dominant, where there are no connections between the superficial and deep venous system on a pre-operative computed tomography angiography (CTA), need venous supercharging [18, 52, 59]. However, there are less clear cases; for example, in cases with atypical connections or in cases where dynamic changes occur when the flap has been raised [15, 18]. Such dynamic changes cannot be predicted from the pre-operative CTA and might not always give rise to immediate intra-operative clinical signs of venous congestion. None of the clinical signs investigated in this review, such as the classical proposal that a SIEV diameter of > 1.5 mm or a high BMI indicate a need for venous supercharging, seem to be valid predictors [9, 50].

Most of the included studies are cohorts comparing patients with clinical signs of venous congestion, and thereby an indication for venous supercharging, with patients without clinical signs of venous insufficiency. Hence, two completely different groups are compared. Although, it has been performed in three studies [31, 35, 58], it is not viable that all patients are randomly allocated to venous supercharging or no venous supercharging, as it will be clinically indicated in some cases and not in others. To randomise patients with clinical signs of venous congestion would be both ethically and scientifically wrong. A solid uncertainty about which treatment alternative (venous supercharging or not venous supercharging) is more beneficial is a fundamental prerequisite to allow for a randomised trial (*theoretical equipoise*) and as most plastic surgeons would agree that clinical venous congestion has to be treated, there is no such general uncertainty in the case of venous supercharging [65]. An alternative could be to randomise only patients without clinical signs of venous congestion, to answer the question if venous supercharging is beneficial in patients without clinical signs of venous congestion. Such a trial would necessitate definitions of complications, such as venous congestion, to enable correct sample size calculations and quantification of the magnitude of the effect.

Another weakness of the included studies, for example in the RCT [31], is that the frequency of venous congestion, total/partial flap loss (Additional file 1) and surgical take-backs (Additional file 5) seems to be higher respective to most high volume centres. Hence, in future studies, it might be useful to include information about the volumes and experience of flaps in the centre where the study is performed.

For many years, the lack of widely accepted standardised definitions and reporting of complications has been a major weakness in surgical research and an obstacle to increase the quality of evidence [66]. Great efforts, such as the work of Clavien and Dindo, have been made to develop and validate classification systems and general definitions of surgical complications, which has advanced evidence based medicine in the surgical fields [67, 68]. The Clavien–Dindo Classification is based on ranking the severity of the intervention required to treat a given complication; for example, grade I is a complication that results in deviations from the postoperative course not requiring the need for pharmacological treatment intervention or surgical/endoscopic/radiological intervention and grade V complications that result in death [67]. Nonetheless, to optimize reconstructive microsurgical techniques, standardised reporting of procedure specific complications, not just the severity of the intervention to treat them, is necessary.

In the present paper, we found that most studies report complication, such as venous congestion, fat necrosis, and partial flap loss, without defining the complication or how and by whom it has been diagnosed (Additional file 1). Fat necrosis is a common complication after abdominally based free flaps [69, 70]. Even so, there is no uniformly accepted definition of how it should be diagnosed or quantified [69], which makes the true frequency as well as studies on how it can be lowered of a low scientific quality. The most common definitions include “palpable subcutaneous firmness not due to cancer,” “firmness measuring 1 or 2 cm in diameter” or an “ultrasound-detectable lesion” [69]. All these definitions contain a substantial part of subjectiveness and have never been validated. In fact, a protocol for how fat necrosis should be diagnosed and quantified with ultrasound has never been described [71], let alone validated/reliability tested. Other complications, such as total flap loss, are, per definition, more standardised. However, total flap loss is a rare adverse event which makes it difficult to use as a single primary outcome in a scientific study comparing different techniques. Moreover, the frequency of complications is affected by both patient related factors, such as comorbidities, BMI, and smoking, as well as surgical factors. Hence, to study surgical factors, for example the effect of venous supercharging, patient-related factors must be controlled and the analyses adjusted for them. In the present review, most included studies did not report patient-related factors, which is a clear weakness. In brief, the complication reporting of the studies included in the present studies makes the result unreliable. It also casts doubt on the validity of the previous reviews where meta-analyses seem to have been performed on unacceptably heterogenic data [72, 73]. To advance the field of abdominally based free flaps and refine our techniques in a scientific manner, as well as enabling comparison of the result from different studies, further studies on standardisation of diagnosing and reporting of specific complication must be performed. In addition, the standardisation must comprise follow-up times, registration of complications, as well as adjustment for risk factors for complications and base line characteristics.

In conclusion, it might be difficult to predict in which in cases where there are no clinical signs of venous congestion. The overall certainty of evidence is very low (GRADE ⊕ ⊝ ⊝ ⊝) for using radiological findings, preoperative measurements, and clinical risk factors to decide whether to use venous supercharging, for the occurrence of donor site complications after SIEV harvesting and for the effect on length of hospital stay and on the operative time. The overall certainty of evidence is low (GRADE ⊕ ⊕ ⊝ ⊝) for the effect of venous supercharging on flap complications and on surgical takebacks. To enable an increase of the scientific evidence, a standardised classification and reporting system of common complications after abdominally based free flaps are needed.

### Supplementary Information

**Additional file 1.** Flap complications.

**Additional file 2.** Donor site complications.

**Additional file 3.** Length of hospital stay (LOS).

**Additional file 4.** Operative time.

**Additional file 5.** Surgical take backs.

**Additional file 6.** Strategies.

**Additional file 7.** PRISMA 2020 Checklist.

## Data Availability

The datasets supporting the conclusions of this article are included with the article and its additional files.

## Abbreviations

BGMI: Blood glucose measurement index
BMI: Body mass index
BV: Basilic vein
C: Comparison
CI: Confidence interval
CLBS: Combined laser Doppler spectrophotometry system
CSV: Circumflex scapular vein
CT: Computer tomography
CTA: Computer tomography angiography
CV: Cephalic vein
DIEA: Deep inferior epigastric artery
DIEP: Deep inferior epigastric perforator flap
DIEV: Deep inferior epigastric vein
DIEVc: *Vena comitantis* of the DIEV
EJV: External jugular vein
GRADE: Grading of Recommendations, Assessment, Development and Evaluations
I: Intervention
ICG: Indocyanine green
IMV: Internal mammary vein/internal thoracic vein
IQR: Interquartile range
LMV: Lateral mammary vein
LOS: Length of hospital stay
LTV: Lateral thoracic vein
MAS: Macrovascular arteriovenous shunt
MDCTA: Multidetector-row computed tomography angiography
MRA: Magnetic resonance angiography
ms: Muscle sparing
MSIEV: The medial branch of the SIEV
NA: Not applicable
NR: Not reported
NS: Non-significant
O: Outcome
P: Population
PICO: Population, Intervention, Comparison, and Outcome
RR: Relative risk
SA: Serratus anterior muscle branch of thoracodorsal vein
SCIV: Superficial circumflex iliac vein
SCIVc: *Vena comitantis* of the SCIV
SIEV: Superficial epigastric vein
SOS: Superficial outside-flap shunt
TAV: Thoracoacromial vein
TDV: Thoracodorsal vein
TRAM: Transverse rectus abdominus muscle flap
VG: Venous graft
