# Supplementary material for: A systematic review of the scientific evidence of venous supercharging in autologous breast reconstruction with abdominally based flaps
Source: World J Surg Oncol. 2023 Dec 4;21:379. doi: 10.1186/s12957-023-03254-9 (PMC10694990; doi:10.1186/s12957-023-03254-9)
Supplement: Supplementary file 1 — Additional file 1. Flap complications. [file 12957_2023_3254_MOESM1_ESM.docx]

# Additional file 1: Flap complications

| **Author**  **Year**  **Country** | | **Study type** | | **Study groups; Intervention and control (n= no. of DIEPs)** | | **Complications** | | | | | | | | **Comments** | |
| --- | --- | --- | --- | --- | --- | --- | --- | --- | --- | --- | --- | --- | --- | --- | --- |
| Ayestaray, 2016, France [1] | | Randomised controlled trial (RCT) | | I3: 29  C: 23 | |  | I3 | | | C | p-value | | | No definition of complications and how they were diagnosed.  The patients in the two groups had similar age (54 years vs. 56 years) and BMI (27 vs. 27), frequency of diabetes (38% vs. 35%), and hypertension (62% vs. 65%) as well as similar vessel diameters. | |
|  |  |  |  |  |  | Venous congestion | 3 (13%) | | | 16 (55%) | 0.001 | | |  |  |
|  |  |  |  |  |  | Fat necrosis | 4 (17%) | | | 14 (48%) | 0.02 | | |  |  |
|  |  |  |  |  |  | Partial flap loss | 2 (9%) | | | 13 (45%) | 0.004 | | |  |  |
|  |  |  |  |  |  | Total flap loss | 0 | | | 5 (17%) | 0.036 | | |  |  |
| Al-Dhamin, Canda 2014 [2] | | Non-randomised study (retrospective) with controls | | I1: 17  C: 31 | |  | I1 | | | C | p-value | | | No definition of complications and how they were diagnosed. | |
|  |  |  |  |  |  | Total flap loss | 0 | | | 1 (3%) | 1.0 | | |  |  |
|  |  |  |  |  |  | Partial flap loss | 1 (6%) | | | 3 (10%) | 1.0 | | |  |  |
|  |  |  |  |  |  | Fat necrosis | 3 (18%) | | | 6 (19%) | 1.0 | | |  |  |
|  |  |  |  |  |  | Wound infection | 2 (12%) | | | 2 (6%) | 0.61 | | |  |  |
|  |  |  |  |  |  | Wound dehiscence | 1 (6%) | | | 2 (6%) | 1.0 | | |  |  |
|  |  |  |  |  |  | Hematoma | 1 (6%) | | | 2 (6%) | 1.0 | | |  |  |
| Al Hindi, 2019, France [3] | | Non-randomised study (retrospective) with controls | | I1: 15  I2: 2  C: 181 | |  | I2 | | | C | | | | No definition of complications and how they were diagnosed. | |
|  |  |  |  |  |  | Partial flap loss | 0 | | | 5.5% | | | |  |  |
|  |  |  |  |  |  | Intervention due to hematoma | 0 | | | 5% | | | |  |  |
| Ali, 2010, Taiwan [4] | | Non-randomised study (retrospective) with controls | | I1: 14  I2: 7  C: 130 | |  | I1 | I2 | | C | | I2 vs C | | No definition of complications and how they were diagnosed.  I1 vs C not compared  I1 vs I2: total flap failure p=0.04, total complication rate p=0.03 | |
|  |  |  |  |  |  | Total flap loss | 0 | 1 (14%) | | 0 | | 0.04 | |  |  |
|  |  |  |  |  |  | Partial flap loss | 1 (7%) | 2 (29%) | | 0 | | 0.01 | |  |  |
|  |  |  |  |  |  | Fat necrosis | 1 (7%) | 2 (29%) | | 8 (6%) | |  | |  |  |
|  |  |  |  |  |  | Total complication rate | 2 (14%) | 5 (71%) | | 29 (22%) | |  | |  |  |
| Boutros, 2013, USA [5] | | Non-randomised study (retrospective) with controls | |  | |  | I1 | | | C | | p-value | | No definition of complications.  Fat necrosis was diagnosed with ultrasound (no further information is given on the methodology). | |
|  |  |  |  | Ultrasound groups:  I3: 63  C:6 | | Clinical and ultrasonic fat necrosis | 4 (6.3%) | | | 1 (17%) | | NS | |  |  |
|  |  |  |  |  |  | Clinical fat necrosis | 2(3.2%) | | | 1 (17%) | |  |  |  |  |
|  |  |  |  | Total groups:  I3: 311  C: 42 | | Total flap failure | 0 | | | 0 | |  | |  |  |
| Enajat, 2010, Australia [6] | | Non-randomised study (retrospective) with controls | | I1: 291  C: 273 | |  | I1 | | | Controls | | I vs. C | | No definition of complications and how they were diagnosed.  Venous thrombosis was not clinically detectable in the SIEV group (identified with implantable Doppler probe), whereas it was in the controls.  There were no cases of venous thrombosis in both veins. One venous thrombosis in the controls resulted in complete flap failure and none in the SIEV group. The cases of complete flap failure were caused by arterial thrombosis. | |
|  |  |  |  |  |  | Venous congestion | 0 | | | 7 (2.6%) | | 0.006 | |  |  |
|  |  |  |  |  |  | Arterial thrombosis | 8 (3%) | | | 10 (4%) | | 0.54 | |  |  |
|  |  |  |  |  |  | Venous thrombosis | 5 (2%) | | | 5 (2%) | | 0.92 | |  |  |
|  |  |  |  |  |  | Total flap loss | 6 (2%) | | | 5 (2%) | | 0.38 | |  |  |
|  |  |  |  |  |  | Partial flap loss | 2 (0.7%) | | | 2 (0.7%) | | 0.98 | |  |  |
|  |  |  |  |  |  | Hematoma | 21 (7%) | | | 23 (8%) | | 0.58 | |  |  |
|  |  |  |  |  |  | Infection | 37 (13%) | | | 23 88%) | | 0.16 | |  |  |
|  |  |  |  |  |  | Fat necrosis | 25 (9%) | | | 23 (11%) | | 0.26 | |  |  |
|  |  |  |  |  |  | Seroma | 9 (3%) | | | 2 (0.7%) | | 0.08 | |  |  |
| Eom, 2011, South Korea [7] | | Non-randomised study (retrospective) with controls | | I1: 45  C: 108 | |  | I1 | | | C | | p-value | | No definition of complications and how they were diagnosed.  The I1 group had higher BMI and more smokers than the controls. | |
|  |  |  |  |  |  | Venous congestion | 0 | | | 9 (8.3%) | | 0.046 | |  |  |
|  |  |  |  |  |  | Venous obstruction | 0 | | | 7 (6.5%) | | 0.080 | |  |  |
|  |  |  |  |  |  | Total flap loss | 0 | | | 2 (1.9%) | | 0.36 | |  |  |
| La Padula, 2016, France [8] | | Non-randomised study (retrospective) with controls | | I1: 36  C: 38 | |  | I1 | | | C | |  | | No definition of complications and how they were diagnosed. | |
|  |  |  |  |  |  | Total flap loss | 0 | | | 1 (2.8%) | | 0.33 | |  |  |
|  |  |  |  |  |  | Partial flap loss | 0 | | | 4 (11%) | | 0.045 | |  |  |
| Lee, 2013, South Korea [9] | | Non-randomised study (retrospective) with controls | | I1: 18  C: 68 | | Fat necrosis 5 patients with SIEV  No fat necrosis 13 patients with SIEV, p=0.194 | | | | | | | | No definition of complications and how they were diagnosed. | |
| Ochoa, 2013, USA [10] | | Non-randomised study (retrospective) with controls | | I1: 87  C: 629 | |  | I1 | | | C | | p-value | | No definition of complications and how they were diagnosed. | |
|  |  |  |  |  |  | Infection | 1.2% | | | 5.4% | | 0.02 | |  |  |
|  |  |  |  |  |  | Hematoma | 1.2% | | | 1.9% | | 0ö6 | |  |  |
|  |  |  |  |  |  | Seroma | 0 | | | 0.8% | | NA | |  |  |
|  |  |  |  |  |  | Fat necrosis | 12.8% | | | 10.4% | | 0.53 | |  |  |
|  |  |  |  |  |  | Delayed wound healing | 10.5% | | | 6.3% | | 0.62 | |  |  |
|  |  |  |  |  |  | Vessel thrombosis | 0 | | | 0.6% | | NA | |  |  |
|  |  |  |  |  |  | Flap failure | 0 | | | 1% | | NA | |  |  |
|  |  |  |  |  |  | Total | 21.8% | | | 23.8% | | 0.37 | |  |  |
| Santanelli, 2015, Italy [11] | | Non-randomised study (retrospective)  with controls | | I1: 173  C:74 | |  | I1 | | | C | |  | | No definition of complications and how they were diagnosed.  The authors concluded “ SIEV is not a risk factor for fat necrosis and partial flap loss” | |
|  |  |  |  |  |  | Complications | 20 (12%) | | | 12 (16%) | | 0.43 | |  |  |
| Tokumoto, 2019, Japan [12] | | Non-randomised study (retrospective) with controls | | I1: 45  C: 43 | |  | I1 | | | C | | p-value | | No definition of complications and how they were diagnosed. | |
|  |  |  |  |  |  | Total flap loss | 1 (2.2%) | | | 0 | | 0.51 | |  |  |
|  |  |  |  |  |  | Partial flap loss | 6 (13%) | | | 10 (23%) | | 0.17 | |  |  |
| Xin, 2012, China [13] | | Non-randomised study (retrospective) with controls | | I1: 32  C: 47 | |  | I1 | | | C | | | | No definition of complications and how they were diagnosed. | |
|  |  |  |  |  |  | Total flap loss | 0 | | | 1 (2.1%) | | | |  |  |
|  |  |  |  |  |  | Partial flap loss | 1 (3%) | | | 4 (8.5%) | | | |  |  |
| Vijayasekaran, 2017, USA [14] | | Non-randomised study (retrospective) with controls | | I3: 30  C: 30 | |  | I3 | | | C | | | | No definition of complications and how they were diagnosed.  The two groups were similar regarding age (48 vs. 49 years), BMI (30.4 vs. 30.1), frequency of diabetes (10% vs. 13%), history of smoking (13% vs. 13%), and previous abdominal surgery (27% vs. 27%). There were more patients with hyperlipidemia in the control group (33% vs. 27%) and more patients with hypertension in the intervention group (27% vs. 20%). | |
|  |  |  |  |  |  | Total flap loss | 0 | | | 0 | | | |  |  |
|  |  |  |  |  |  | Fat necrosis | 1 (3%) | | | 3 (10%) | | | |  |  |
| Bartlett, 2018, USA [15] | Case series (retrospective) | | I1: 67  C: 172 | |  | | I1 | | |  | | p-value | | | Major complications were “any events necessitating an unplanned return t the operating room”. The authors also state that all complications “regardless of severity” were recorded. Complications and how they were diagnosed were not otherwise defined. |
|  |  |  |  |  | Complications | | 6 (16%) | | | 13 (13%) | | p=0.90 | | |  |
| Unukovych, 2016, Sweden [16] | Case series (retrospective) | | I1: 211  C: 292 | |  | | I1 | | | C | | p-vaule | | | Complications were “any unanticipated event requiring return to the operating room and general anesthesia.”  As corrections and elective operations were included in the re-operations, the data is given in this table rather than in the table on takebacks. |
|  |  |  |  |  | Reoperation (n=80) | | 24 (30%) | | | 56 (70%) | | 0.043 | | |  |
|  |  |  |  |  | No reoperation (n=423) | | 187 (44%) | | | 236 (56%) | |  | | |  |
| Varnava, 2023, Germany [17] | | Non-randomised study (retrospective) with controls | | I1: 4  C: 146 | |  | I1 | | C | | | | p-value | No definition of complications and how they were diagnosed. | |
|  |  |  |  |  |  | Total flap loss | 4 (2.7) | | 0 | | | | 1 |  |  |
|  |  |  |  |  |  | Partial flap loss | 6 (4.1) | | 0 | | | | 1 |  |  |
|  |  |  |  |  |  | Fat necrosis | 20 (13.7) | | 1 (25) | | | | 1 |  |  |
|  |  |  |  |  |  | Anastomosis revision | 7 (4.8) | | 2 (50) | | | | 0.018 |  |  |
