# Supplementary material for: A systematic review of the scientific evidence of venous supercharging in autologous breast reconstruction with abdominally based flaps
Source: World J Surg Oncol. 2023 Dec 4;21:379. doi: 10.1186/s12957-023-03254-9 (PMC10694990; doi:10.1186/s12957-023-03254-9)
Supplement: Supplementary file 2 — Additional file 2. Donor site complications. [file 12957_2023_3254_MOESM2_ESM.docx]

Additional file 2: Donor site complications

| **Author**  **Year**  **Country** | **Study type** | **Study groups; Intervention and control (n= no. of DIEPs)** | **Results** | | | | | **Comment** |
| --- | --- | --- | --- | --- | --- | --- | --- | --- |
| Nedomansky, 2018, Austria [18] | Non -randomised study (retrospective) with controls | I1: 39  29 unilateral SIEVs  10 bilateral SIEVs  C: 61 |  | Unilat SIEV | Bilat SIEV | Controls |  | Drainage requiring seroma formation was identified with ultrasound |
|  |  |  | Seroma formation | 5 (17%) | 4 (40%) | 7 (11.5%) | Unilat vs. controls p=0.45  Bilat vs. controls p=0.02 |  |
| Svee, 2023, Sweden [19] | Non-randomised study (retrospective) with controls | I1: 27  C: 27 | The usage of the CV does not increase the long-term risk of ipsilateral arm lymphoedema or volume increase. | | | | |  |
| Tokumoto, 2019, Japan [12] | Non-randomised study (retrospective) with controls | I1: 45  Recipient vessels:  22 SA  16 LTV  7 CV | No donor site morbidity where the SIEV was harvested and no morbidity when the recipient vessel was dissected | | | | |  |
