# Supplementary material for: A systematic review of the scientific evidence of venous supercharging in autologous breast reconstruction with abdominally based flaps
Source: World J Surg Oncol. 2023 Dec 4;21:379. doi: 10.1186/s12957-023-03254-9 (PMC10694990; doi:10.1186/s12957-023-03254-9)
Supplement: Supplementary file 3 — Additional file 3. Length of hospital stay (LOS). [file 12957_2023_3254_MOESM3_ESM.docx]

Additional file 3: Length of hospital stay (LOS)

| **Author**  **Year**  **Country** | **Study type** | **Study groups; Intervention and control (n= no. of DIEPs)** | **Length of hospital stay (days)** | | | | | | | | | | | |
| --- | --- | --- | --- | --- | --- | --- | --- | --- | --- | --- | --- | --- | --- | --- |
| Al Hindi, 2019, France [3] | Non-randomised study (retrospective) with controls | I1: 15  I2: 2  C: 181 | I | | | I1 | | | I2 | | | C | | |
|  |  |  | Mean 9 | | | 7.8 | | | 13.5 | | | 7 | | |
| Ali, 2010, Taiwan, USA [4] | Non-randomised study (retrospective) with controls | I1: 14  I2: 7  C: 130 | I1 | | | | I2 | | | | C | | | |
|  |  |  | Mean 11.5±1.8 | | | | 18.2±4.6 | | | | 12.2±4.1 | | | |
| Nedomansky, 2018, Austria [18] | Non -randomised study (retrospective) with controls | I1: 39 (29 unilateral, 10 bilateral)  C: 61 | Unilat I1 | | Bilat I1 | | | C | | Unilat vs. C | | | Bilat vs. C | |
|  |  |  | Mean 13.5±2.5, Median 13 (Range 10-20) | | 13.1±1., 13.5 (11-15) | | | 13.2±2.9, 13(9-28) | | p=0.32 | | | p=0.5 | |
| La Padula, 2016, France [8] | Non-randomised study (retrospective) with controls | I1: 36  C: 38 | I1 | | | | | | C | | | I1 vs. C | | |
|  |  |  | Mean 8±1.58 | | | | | | 9.8±5.7 | | | <0.0001 | | |
| Ochoa, 2013, USA [10] | Non-randomised study (retrospective) with controls | I1: 87 DIEPs (81 pats)  C: 629 DIEPs (418 pats) | I1 -VG | I1 + VG | | | C | | I1 vs. C | | I1 -VG vs. C | | | I1 +VG vs. C |
|  |  |  | Mean 4.8 | 6.2 | | | 4 | | p<0.01 | | p<0.01 | | | p<0.01 |
