# Supplementary material for: A systematic review of the scientific evidence of venous supercharging in autologous breast reconstruction with abdominally based flaps
Source: World J Surg Oncol. 2023 Dec 4;21:379. doi: 10.1186/s12957-023-03254-9 (PMC10694990; doi:10.1186/s12957-023-03254-9)
Supplement: Supplementary file 4 — Additional file 4. Operative time. [file 12957_2023_3254_MOESM4_ESM.docx]

Additional file 4: Operative time

| **Author**  **Year**  **Country** | **Study type** | **Study groups; Intervention and control (n= no. of DIEPs)** | **Operation time** | | | | | | **Comments** |
| --- | --- | --- | --- | --- | --- | --- | --- | --- | --- |
| Al Hindi, 2019, France [3] | Non-randomised study (retrospective) with controls | I1: 15  I2: 2  C: 181 | Mean increase in operative time was 1 hour and 45 minutes in case of secondary SIEV (I2) | | | | | | Mean operative time overall: 5 hours and 56 minutes. Time to perform SIEV NR. |
| Ayestaray, 2016, France [1] | RCT | I3: 29  C: 23 | The mean operative time was 510 minutes (range, 405–590) in the I3 group and 405 minutes (range, 355–460) in the C group, (P < 0.001). | | | | | |  |
| Bartlett, 2018, USA [15] | Non-randomised study (retrospective)  with controls | I1: 67 DIEPs (38 pats)  C: 172 DIEPs (100 pats) | No difference between Is and Cs (p=0.555) | | | | | |  |
| Enajat, 2010, Australia [6] | Non-randomised study (retrospective) with controls | I1: 291  C: 273 | I1: 385 min  C: 383 min, p=0.57 | | | | | |  |
| Ochoa, 2013, USA [10] | Non-randomised study (retrospective) with controls | I1: 87 (81 pats)  C: 629 (418 pats) | Min | I1 | | C | | p-value |  |
|  |  |  | Unilateral  Bilateral  Total | 314  434  413 | | 253  413  339 | | <0.01  <0.01  <0.01 |  |
| Xin, 2012, China [13] | Non-randomised study (retrospective) with controls | I1: 32  C: 47 | I2: 6.6 ± 0.7 hours  C: 6.1 ± 1.2 hours, p<0.05 | | | | | |  |
| Tokumoto, 2019, Japan [12] | Non-randomised study (retrospective) with controls | I3: 45 (prophylactic)  C: 43 | I3 | | C | | p-value | |  |
|  |  |  | 600.6 min ± 71.7 | | 582.7 min ± 61.4 | | 0.10 | |  |
