# Supplementary material for: A systematic review of the scientific evidence of venous supercharging in autologous breast reconstruction with abdominally based flaps
Source: World J Surg Oncol. 2023 Dec 4;21:379. doi: 10.1186/s12957-023-03254-9 (PMC10694990; doi:10.1186/s12957-023-03254-9)
Supplement: Supplementary file 5 — Additional file 5. Surgical take backs. [file 12957_2023_3254_MOESM5_ESM.docx]

Additional file 5: Surgical take backs

| **Author**  **Year**  **Country** | | **Study type** | | **Study groups; Intervention and control (n= no. of DIEPs)** | | **Re-exploration** | | | | | **Comments** |  |
| --- | --- | --- | --- | --- | --- | --- | --- | --- | --- | --- | --- | --- |
| Ayestaray, 2016, France [1] | | RCT | | I3: 29  C: 23 | | I3 | C | |  | |  |  |
|  |  |  |  |  |  | 3 (13%) | 16 (55%) | |  |  |  |  |
| Boutros, 2013, USA [5] | | Non-randomised study (retrospective) with controls | | I3: 311  C: 42 | | I3 | C | | p-value | |  |  |
|  |  |  |  |  |  | 1 (0.3%) | 2 (4.9%) | | 0.0029 | |  |  |
| Eom, 2011, South Korea [7] | | Non-randomised study (retrospective) with controls | | I2: 45  C: 108 | | I2 | C | | p-value | |  |  |
|  |  |  |  |  |  | 1 (2.3%) | 8 (7.4%) | | 0.12 | |  |  |
| La Padula, 2016, France [8] | | Non-randomised study (retrospective) with controls | | I2: 36  C: 38 | | I2 | C | | p-value | |  |  |
|  |  |  |  |  |  | 0 | 5 (14%) | | 0.0242 | |  |  |
| Tokumoto, 2019, Japan [12] | | Non-randomised study (retrospective) with controls | | I3: 45 (prophylactic)  C: 43 | | I3 | C | | p-value | |  |  |
|  |  |  |  |  |  | 2 (4.4%) | 1 (2.3%) | | 0.51 | |  |  |
| Vijayasekaran, 2017, USA [14] | | Non-randomised study (retrospective) with controls  *Two consecutive*  *series* | | I3: 30  C: 30 | | I3 | C | | | |  |  |
|  |  |  |  |  |  | 0 | 2 (6.7 %) | | | |  |  |
| Varnava, 2023, Germany [17] | Non-randomised study (retrospective) with controls | | I1: 4  C: 146 | | I1 | | | C | |  | | |
|  |  |  |  |  | 2 (50) | | | NR | |  |  |  |
