# Supplementary material for: A systematic review of the scientific evidence of venous supercharging in autologous breast reconstruction with abdominally based flaps
Source: World J Surg Oncol. 2023 Dec 4;21:379. doi: 10.1186/s12957-023-03254-9 (PMC10694990; doi:10.1186/s12957-023-03254-9)
Supplement: Supplementary file 6 — Additional file 6. Strategies. [file 12957_2023_3254_MOESM6_ESM.docx]

Additional file 6: Strategies

| **Author**  **Year**  **Country** | |  | **Study groups; Intervention and control (n= no. of DIEPs)** | | **Strategy** | | | | | | | **Comments** | | | | |
| --- | --- | --- | --- | --- | --- | --- | --- | --- | --- | --- | --- | --- | --- | --- | --- | --- |
| **Pre-operative radiological findings** | | | | | | | | | | | | | | | | |
| Bast, 2016 [20] | | Case series | P2: 50 women (100 hemiabdomens) | | CTA  Suprascarpal fat pad thicker than 23 mm had larger SIEVs irrespective of the number of deep system perforators. The authors recommend that a prophylactic SIEV is anastomosed in all patients with a suprascarpal fat pad thickness of > 23 mm. | | | | | | | The study looked at number of perforators, SIEV diameter, and fat pad thickness above and below Scarpa's fascia. | | | | |
| Davis, 2018, UK [21] | Non-randomised study (prospective) with controls | | | I2+3: 13  C:25 | CTA  The authors suggested a classification of venous connections between the superficial and the deep system to risk stratify patients. Type I: normal (≥1 venous connection present on every CT slice between the systems), Type II: absent, Type III: atypical (in terms of (1) calibre, (2) tortuosity, or (3) superficial path).  The clinically congested flaps had more atypical venous connection between the deep and the superficial system (67% vs. 8%), that is the risk for venous congestion is five times as high with a type III connection.  The authors conlcude that patients with type I och II connections are good condidates for DIEP flaps whereas caution should be taken with patients with type III connections. | | | | | | |  | | | | |
| Dortch, 2018, USA [22] | Non-randomized study (retrospective) with controls | | | I1: 29  C: 73 | MRA (Ferumoxytol-enhanced)  The flaps that needed venous augmentation had a bigger SIEV diameter, DIEV diameter and SIEV/DIEV diameter ratio. | | | | | | | The strategy has not been tested scientifically/validated | | | | |
|  |  |  |  |  |  | I1 | | C | | p-value | |  |  |  |  |  |
|  |  |  |  |  | SIEV diameter at primary branch point, mm  SIEV diameter at origin, mm  Ratio of SIEV to DIEV diameter at origin  Ratio of SIEV diameter at origin  to diameter of dominant perforator | 4.1 ± 0.9  4.0 ± 0.9  1.05 ± 0.3  1.6 ± 0.5 | | 3.7 ± 0.8  3.6 ± 0.7  0.93 ± 0.2  1.3 ± 0.3 | | 0.01  0.04  0.03  0.001 | |  |  |  |  |  |
| Zhu, 2023, Republic of Korea [23] | Case series (retrospective) | | P1: 62 ms-TRAMs, 6 DIEPs | | Indocyanine green and CTA  The flaps were categorized into three groups based on indocyanine green findings post SIEV anastomosis:   - Group 1: >3% decrease in hypoperfused area (n=42) - Group 2: change in hypoperfused area ranging from −3% to 3% (n=20) - Group 3: >3% increase in hypoperfused area (n=6)   When the pre-operative CTAs were reviewed it was found that there was a higher mean number of midline-crossing branches and bigger SIEV diameter (p-value 0.002 and 0.039 respectively) in group 1 than in the other two groups.  The authors recommend that the SIEV is anastomosed when the contralateral SIEV has more than two midline-crossings or when the diameter contralateral SIEV is greater than on the pedicled side. | | | | | |  | | | | | |
| Huang, 2022, USA [24] | Non-randomised study (retrospective) with controls | | I1: 5  I2: 3  C: 182 | | CTA  The SIEV diameter (mean 2.5 ±0.6 mm) was positively correlated with suprascarpal fat pad thickness (r = 0.51, p<0.001) and total abdominal fat pad thickness (r=0.51, p<0.001)  Congested flaps had significantly thinner suprascarpal fat pads (4.0 vs. 6.0 mm, p=0.030) and total abdominal fat pads (16.2 vs. 26.0 mm, p=0.037) than controls  All congested flaps in the series had a suprascarpal fat pad thickness of <18mm. Among the controls 54% (182/335) had a thickness of <18mm (OR 10.9)  The authors recommend that a prophylactic SIEV is anastomosed in all patients with a suprascarpal fat pad thickness of < 18 mm. | | | | | | | |  | | | |
| Katz, 2010, USA [25] | Case series (prospective) | | P4: 172 hemiabdomens (86 pats) | | CTA  The authors recommend that a SIEV is anastomosed in patients with type IV anatomy (superficial dominant) on CTA. They define superficially dominant as there are CTA signs that the SIEV that is ‘seemingly adequate to support transferred abdominal tissue’. | | | | | | | |  | | | |
| Sadik, 2013, USA [26] | Non-randomised study (prospective) with controls | | I1: 6  C: 33 | | CTA  There was no correlation between the requirement of a SIEV anastomosis and   - preoperative CT diamenter of SIEV (Spearman’s correlation coefficient 0.25) | | | | | | | | |  | | |
| Schaverien, 2010, UK [27] | Non-randomized study (retrospective) with controls | | P1: 54 DIEPs | | MRA (contrast-enhanced)  There is a risk of venous congestion if no direct connection can be seen between venea commitantes and SIEV on MRA. | | | | | | | | |  | | |
|  |  |  |  |  |  | | Direct connection perforator venae comitantes to SIEV | | No direct connection perforator venae comitantes to SIEV | | | | |  |  |  |
|  |  |  |  |  | Venous congestion  No venous congestion | | 0  46 | | 7  1 | | | | |  |  |  |
| Wagels, 2015, Australia [28] | Non-randomized study (retrospective) with controls | | P1: 124 DIEPs/TRAMs (96 pats) | | CTA  The authors found the following signs predictive of venous congestion:   - SIEV size >DIEV size at origin (5.2 vs 3.5 mm, p = 0.007) - an axial non-arborising superficial system (96.7% vs 0, p < 0.001) - superficial system not connected to deep system (38.1 vs 88.8%, p < 0.001) - a type I pedicle* (75 vs 64.2%, p = 0.22) | | | | | | | | |  | | |
| **Intraoperative measurements** | | | | | | | | | | | | | | | | |
| Akita, 2018, Japan [29] | Caser series (prospective) | | I4: 8 DIEPs with BGMI<0.8 | | Blood glucose measurement index (BGMI), the ratio of blood glucose content in the flap to systemic blood glucose, can be used after wound closure to identify venous congestion requiring a SIEV anastomosis.  SIEV anastomisis was performed in patient with BGMI<0.8. Average BGMI improved from 0.71±0.05 to 0.94±0.05 (P < .01) after anastomisis. There were no complications in the patients. | | | | | | | | |  | | |
| Beier, 2013, German [30] | Case series (prospective) | | P1: 25 DIEPs or muscle sparing TRAMs | | Combined laser Doppler spectrophotometry system (CLBS) influence on decision making:  3/25 moderate influence, the decision was difficult for the surgeon, but strongly supported by CLDS finding.  2/25 significant influence  20/25 no influence (DLDS confirmed the clinical findings) | | | | | | | | |  | | |
| Rothenberger, 2013, Germany [31] | Case series (prospective) | | P1: 19 DIEPs | | Intermittent clamping. Venous congestion was quantified intraoperatively with relative HB concentration (spectrometric technique).  There was a significant increase in the venous drainage only in the contralateral zones (II and IV) after declamping of SIEV  The authors conclude that an extra SIEV anastomosis might be beneficial particularly in flaps with large portions of the contralateral zones | | | | | | | | | |  | |
| Smit, 2010, Sweden (multicentre) [32] | Case series (prospective) | | P1: 26 DIEPs | | Mean increase in pressure between measurement at the beginning of dissection and after the raise on a single perforator 10.6 mm Hg (μ = 10.6; range −1 to 31; Ó ± 7.0 mm Hg). One case of clinical venous congestion was seen and in that case the increase in pressure was 31 mm Hg, which was the highest in the series. | | | | | | | | | | |  |
| **Clinical signs** | | | | | | | | | | | | | | | | |
| Blondeel, 2000, Belgium [33] | Non-randomised study (retrospective) with controls | | I1: 5  C: 245 | | The authors suggest that if a SIEV of >1.5 mm is present the SIEV should be preserved for possible used in salvage as this could indicafe a dominant superficial system. | | | | | | | | | | |  |
| Galanis, 2014, USA [34] | Description of centre’s approach | | NA | | The authors suggest that venous augmentation is performed if the flap is globally congested. If the flap is focally congested flap thinning should be considered. | | | | | | | | | | |  |
| Lundberg, 2006, Sweden [35] | Non-randomised study (retrospective) with controls | | P1:3  P2: 3  C:44 | | The authors recommend that a prophylactic SIEV is anastomosed | | | | | | | | | | |  |
| Ochoa, 2013, USA [10] | Non-randomised study (retrospective) with controls | | I1: 87 DIEPs (81 pats)  C: 629 DIEPs (418 pats) | | Risk factors for requiring venous augmentation:  Increasing number of perforators OR: 1.46 (1.17-1.83), p= 0.004  Previous abdominal surgery OR: 0.8 (0.66-0.99), p=0.03  Body mass index OR: 10.4 (0.99-1.10), p=0.14 | | | | | | | | | | |  |
| Rubino, 2009, Italy [36] | Case series (prospective) | | P1: 19 DIEPs | | The bigger the flap the bigger the flow rate and therefore the need for a higher venous drainage. Two ways to increase the total cross section area is to add veins in parallel and choose larger diameter veins.  Flow rate was understood from an equation derived from regression analysis: Flow (ml/s) = 0.001xflap weight (grams)-0.04 | | | | | | | | | | |  |
| Sadik, 2013, USA [26] | Non-randomised study (prospective) with controls | | I1: 6  C: 33 | | There was no correlation between the requirement of a SIEV anastomosis and   - In situ diamenter of SIEV (Spearman’s correlation coefficient 0.06) - Age - BMI - BMI:SIEV size ratio | | | | | | | | | | |  |
| Tokumoto, 2019, Japan [12] | Non-randomised study (retrospective) with controls | | I3: 45 (prophylactic)  C: 43 | | The authors supercharge the flap if the DIEV≤IMV in combination of clinical signs of venous congestion after anastomosis or if the DIEV≥SIEV in combination of a bleeding SIEV after anastomosis. | | | | | | | | | | | In the group DIEV≤IMV 54.5% of the flaps showed signs of venous congestion after anastomosis. In the group DIEV≥SIEV 96.6% of the SIEVs were bleeding after anastomosis. |
| Vijayasekaran, 2017, USA [14] | Non-randomised study (retrospective) with controls | | I3: 30  C: 30 | | The authors conclude that the only downside with prophylactic usage of SIEV is an increase of operative time of 20 minutes and the cost of an extra COUPLER^©^ device (Synovis Micro Companies Alliance, Inc. Birmingham, AL, USA) | | | | | | | | | | |  |
